# White matter integrity and processing speed in sickle cell anemia

**DOI:** 10.1212/WNL.0000000000005644

**Published:** 2018-06-05

**Authors:** Hanne Stotesbury, Fenella J. Kirkham, Melanie Kölbel, Philippa Balfour, Jonathan D. Clayden, Sati Sahota, Simrat Sakaria, Dawn E. Saunders, Jo Howard, Rachel Kesse-Adu, Baba Inusa, Maria Pelidis, Subarna Chakravorty, David C. Rees, Moji Awogbade, Olu Wilkey, Mark Layton, Christopher A. Clark, Jamie M. Kawadler

**Affiliations:** From Developmental Neurosciences (H.S., F.J.K., M.K., P.B., J.D.C., S. Sahota, S. Sakaria, C.A.C., J.M.K.), UCL Great Ormond Street Institute of Child Health, London; University Hospital Southampton NHS Foundation Trust (F.J.K.); Clinical and Experimental Sciences (F.J.K.), University of Southampton; Department of Radiology (D.E.S.), Great Ormond Street Hospital NHS Foundation Trust, London; Department of Haematology and Evelina Children's Hospital (J.H., R.K.-A., B.I., M.P.), Guy's and St Thomas' NHS Foundation Trust, London; King's College Hospital NHS Foundation Trust (S.C., D.C.R., M.A.), London; North Middlesex University Hospital NHS Foundation Trust (O.W.), London; and Department of Haematology (M.L.), Imperial College Healthcare NHS Foundation Trust, London, UK.

## Abstract

**Objective:**

The purpose of this retrospective cross-sectional study was to investigate whether changes in white matter integrity are related to slower processing speed in sickle cell anemia.

**Methods:**

Thirty-seven patients with silent cerebral infarction, 46 patients with normal MRI, and 32 sibling controls (age range 8–37 years) underwent cognitive assessment using the Wechsler scales and 3-tesla MRI. Tract-based spatial statistics analyses of diffusion tensor imaging (DTI) and neurite orientation dispersion and density imaging (NODDI) parameters were performed.

**Results:**

Processing speed index (PSI) was lower in patients than controls by 9.34 points (95% confidence interval: 4.635–14.855, *p* = 0.0003). Full Scale IQ was lower by 4.14 scaled points (95% confidence interval: −1.066 to 9.551, *p* = 0.1), but this difference was abolished when PSI was included as a covariate (*p* = 0.18). There were no differences in cognition between patients with and without silent cerebral infarction, and both groups had lower PSI than controls (both *p* < 0.001). In patients, arterial oxygen content, socioeconomic status, age, and male sex were identified as predictors of PSI, and correlations were found between PSI and DTI scalars (fractional anisotropy *r* = 0.614, *p* < 0.00001; *r* = −0.457, *p* < 0.00001; mean diffusivity *r* = −0.341, *p* = 0.0016; radial diffusivity *r* = −0.457, *p* < 0.00001) and NODDI parameters (intracellular volume fraction *r* = 0.364, *p* = 0.0007) in widespread regions.

**Conclusion:**

Our results extend previous reports of impairment that is independent of presence of infarction and may worsen with age. We identify processing speed as a vulnerable domain, with deficits potentially mediating difficulties across other domains, and provide evidence that reduced processing speed is related to the integrity of normal-appearing white matter using microstructure parameters from DTI and NODDI.

Even in the absence of silent cerebral infarction (SCI), patients with sickle cell anemia (SCA) are at risk of cognitive impairment that may worsen with age^1,2^ and affect quality of life.^3^ Reduced processing speed is the most prominent impairment^4^ and may mediate difficulties across other domains,^5^ but the etiology is not well understood, and there are no models of risk factors.

MRI studies have revealed hemodynamic^6–11^ and structural abnormalities^12–14^ that may underlie cognitive impairment. Diffusion tensor imaging (DTI) studies have reported widespread reductions in fractional anisotropy (FA) and increases in radial diffusivity (RD).^15–17^ Diffusion changes have been associated with oxygen desaturation and anemia^15^ and may relate to reduced processing speed, but functional consequences have yet to be investigated.

A limitation of DTI is that the parameters are not specific to particular microstructural elements of white matter. Neurite orientation dispersion and density imaging (NODDI)^18^ may offer more sensitivity and specificity as it models changes in fiber dispersion (orientation dispersion index [ODI]) as well as density of the tissue microstructure (intracellular volume fraction [ICVF]). NODDI has been successfully applied in studies of typical development^19,20^ and clinical populations^21^ but not yet in SCA.

In the present study, we aimed to (1) investigate differences in processing speed between patients with SCA, grouped by presence of SCI, and controls; (2) explore the effect of processing speed on general intelligence and potential risk factors for deficits; and (3) examine the relationship between DTI and NODDI-derived indices of white matter microstructure and processing speed.

## Methods

### Patients

Patients were aged 8 to 38 years and enrolled in 2 studies at University College London: the Sleep and Asthma Cohort follow-up study (SAC-III)^22^ and Prevention of Morbidity in SCA 2b (POMS)^23^ baseline investigation. Controls were healthy siblings of patients recruited to either study with no history of neurologic or psychiatric conditions. Participants were recruited and assessed between 2015 and 2016. Patients were ineligible for SAC and POMS study participation if they were receiving nocturnal respiratory support at the time of enrollment, participating in a clinical trial evaluating blood transfusion or oxygen therapy, or had chronic lung disease (other than asthma) or existing respiratory failure. Additional exclusion criteria for the POMS study were hospital admissions for acute sickle complications within 1 month of enrollment, more than 6 hospital admissions for acute sickle complications within 12 months of enrollment, overnight oximetry showing mean overnight saturation of less than 90% for more than 30% of total sleep time, severe sleep apnea defined by 4% oxygen desaturation index >15/h, and chronic blood transfusion or transfusion within 3 months of enrollment. For the SAC study, patients were enrolled without regard to past sickle- or sleep-related morbidity or transfusion status.

### Standard protocol approvals, registrations, and patient consents

Ethical approval was granted by West London and South Yorkshire research ethics committees, respectively. Full informed consent and assent according to the Declaration of Helsinki were obtained from participants and for children from their parent/guardian.

### Cognitive variables

Full Scale IQ (FSIQ) was measured using the Wechsler Abbreviated Scale of Intelligence (WASI-II subscale IQ; POMS patients), Wechsler Intelligence Scale for Children (WISC-IV; SAC patients and controls younger than 16 years), or the Wechsler Adult Intelligence Scale (WAIS-IV; SAC patients and controls 16 years or older). Processing speed index (PSI) was derived from the WISC-IV or the WAIS-IV using the coding and symbol search subtests. Strong correlations have been demonstrated between editions (WASI/WAIS/WISC) and between the child and adult versions (WISC/WAIS), justifying their inclusion in the same analyses.^24,25^ Assessments were double-scored by trained assessors (J.M.K., M.K., H.S., P.B.) that were blinded to disease status. In the event of disagreement or ambiguity, the opinion of a third assessor was sought.

### Socioeconomic variables

Education decile was obtained from UK postcode to provide an index of socioeconomic status (SES).^26^ This scale captures attainment and skills in local areas based on several indicators: average scores for pupils in state-funded schools at ages 7–11 and 14–16 years, absence from state-funded secondary schools, proportion of people staying on in education/training post 16 years, entry to higher education, proportion of working adults with no/low qualifications and language proficiency. Total scores are ranked from 1 to 10, with 1 representing the most deprived.

### Hematologic variables

Steady-state hemoglobin was recorded from patient medical records using the closest available full blood within 6 months of the day of cognitive testing. Arterial oxygen content (Cao_2_) was calculated using: 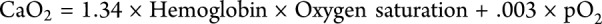
where oxygen saturation (Spo_2_) was estimated by pulse oximetry on the day of cognitive testing (SAC) or the baseline clinic visit (POMS), and po_2_, the partial pressure of oxygen, was assumed to be 100 torr in room air.

### MRI acquisition

Imaging was conducted within 2 weeks of cognitive assessment on a 3T Siemens Prisma (Erlangen, Germany) with 80 mT/m gradients and a 64-channel receive head coil. The MRI protocol included axial T2-weighted (repetition time [TR] = 8,420 milliseconds [ms], echo time [TE] = 68 ms, voxel size = 0.51 × 0.51 × 5.6 mm), fluid-attenuated inversion recovery (FLAIR) (TR = 5,000 ms, TE = 395 ms, voxel size = 0.65 × 1 × 0.65 mm), and diffusion-weighted (TR = 3,050 ms, TE = 60 ms, 2 shells at b = 1,000 s/mm^2^ and b = 2,200 s/mm^2^ with 13 interleaved b = 0 images, voxel size = 2 × 2 × 2 mm) sequences. A neuroradiologist (D.S.), blinded to disease status, read each participant's MRI and classified SCI according to the criteria of a hyperintensity on FLAIR of more than 3 mm in diameter and present on 2 planes, as for the Silent Infarction Transfusion trial.^27^

### MRI processing

The diffusion images were preprocessed using TractoR 3.0.7^28^ and FSL 5.0.1.^29^ Images were visually screened for motion and corrected for susceptibility-induced distortions and eddy current artifact using FSL. Maps for each of the DTI parameters were generated in FSL by fitting a diffusion tensor model to each voxel using a weighted least-squares method. ODI and ICVF maps were generated using the NODDI MATLAB Toolbox.^18^ DTI and NODDI parameters were analyzed using whole-brain, voxel-wise tract-base spatial statistics. The specifics of this approach have been described elsewhere.^30^ Briefly, each participant's FA map was aligned with every other FA map, and the most representative map was used as the target. The target was affine-aligned to Montreal Neurological Institute standard space. All FA maps underwent nonlinear transformation to the target and affine transformation to standard space. FA maps were merged, and voxels with the highest FA at the core of main white matter tracts (threshold: FA = 0.2) were used to create a mean FA skeleton. Each participant's FA map was projected onto the mean FA skeleton, enabling voxel-wise statistical analyses. The maps for the remaining parameters were similarly projected onto the skeleton for analyses. Reference was made to the JHU (Johns Hopkins University) DTI white matter atlas to describe the locations of significant voxels.^31^

### Statistical analysis

Analyses were performed in RStudio Desktop 1.0.153 using the companion to applied regression^32^ and global validation of linear models^33^ packages. Prior to statistical analysis, neurocognitive variables were assessed for normality and equality of variance using the Shapiro-Wilk and Levene tests, respectively. For all analyses, results were considered significant at *p* < 0.05. FSIQ and PSI were compared between patient and sibling control groups using type II analyses of covariance including education deciles as covariates. The effect of PSI on other domains of cognition was explored by including PSI as a covariate in comparisons between patients and controls in FSIQ. An exploratory multiple linear regression analysis was performed to predict PSI from previously implicated and potentially confounding variables: presence of SCI (SCI+/−), Cao_2_, education decile, age, sex, hydroxyurea use, and transfusion status. Influential measures were assessed by calculating the standardized difference of the β for each model variable, difference in fits, covariance ratios, Cook distances, and the diagonal elements of the hat matrix. For all patients, intersubject voxel-wise correlation was performed between DTI and NODDI parameters and PSI, while treating age, sex, and postcode-based education deciles as covariates. Threshold-free cluster enhancement was used to correct for multiple comparisons.

### Data availability

Full anonymized data will be shared at the request from any qualified investigator. Interactive maps from imaging analyses will be uploaded on neurovault where results can be explored and downloaded (neurovault.org/collections/3510/).

## Results

### Patient characteristics

**Figure 1:**
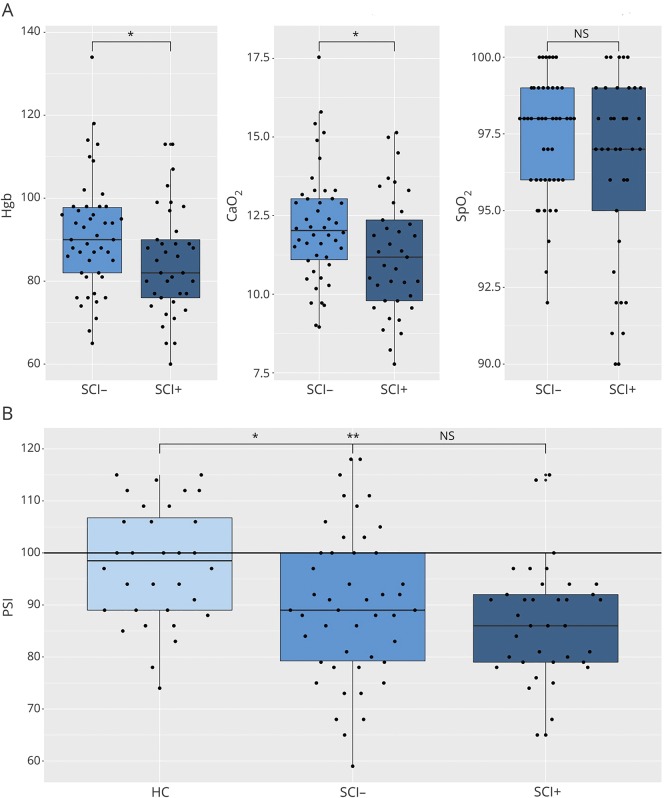
Neurocognitive and hematologic variables (A) Differences in hemoglobin (Hgb; left), arterial oxygen content (Cao_2_; middle), and oxygen saturation (Spo_2_), between patients with (SCI+) and without (SCI−) silent cerebral infarction. (B) Differences in processing speed index (PSI) between healthy controls (HCs) and patients (SCI−, SCI+). *p < 0.05; **p < 0.01 (after Bonferroni correction for multiple comparisons). Horizontal line represents mean PSI in the normative population. NS = not significant.

There were no differences between groups in age or sex (table 1). Of 83 patients (82 sickle cell hemoglobin, 1 hemoglobin S/β^0^-thalassemia), 37 (45%) without neurologic signs were identified with SCI (SCI+). Lesions were right-sided in 8 patients, left-sided in 6, and bilateral in 23. Thirteen patients had lesions in more than one region; 35 had frontal lesions, 12 had parietal lesions, 2 had temporal lesions, and 3 had occipital lesions. Lesions were most frequently located in the border zones between arterial distributions in the deep frontal white matter. Twenty-nine patients were on hydroxyurea (16 SCI−), 5 were on chronic transfusions (3 SCI−), and 9 had undergone a transfusion within 6 months of assessment (6 SCI−). Mean hemoglobin, Spo_2_, and Cao_2_ were lower than reference norms (table 1). Eighteen patients (22%, 8 SCI−) were desaturated with Spo_2_ ≤96%. Mean hemoglobin and Cao_2_ were lower in patients with SCI, but there were no differences in saturation (figure 1). This pattern remained when transfused patients were excluded.

**Table 1:**
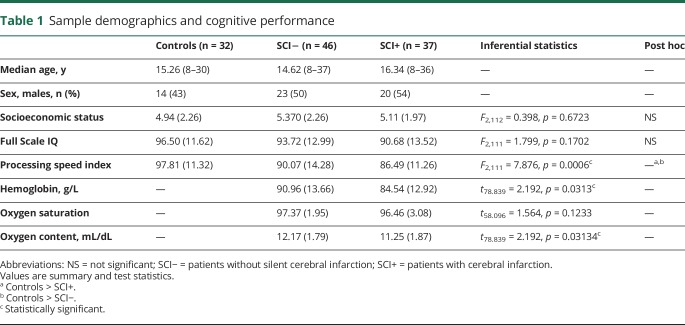
Sample demographics and cognitive performance

### Neurocognitive variables

The data met the analyses of covariance and multiple linear regression assumptions. After controlling for the effect of education deciles, mean PSI was lower in patients than controls by 9.34 scaled points (95% confidence interval: 4.635–14.855, *p* = 0.0003). FSIQ was lower by 4.14 scaled points (95% confidence interval: −1.066 to 9.551, *p* = 0.1), but this difference was abolished when PSI was included as a covariate (*p* = 0.18). There were no differences in cognition between patients with and without SCI, and both groups had lower PSI (table 1; *p* < 0.001) but not FSIQ, compared to controls (figure 1). This pattern remained when analyses were repeated in children and adults separately (*p* < 0.05).

**Figure 2:**
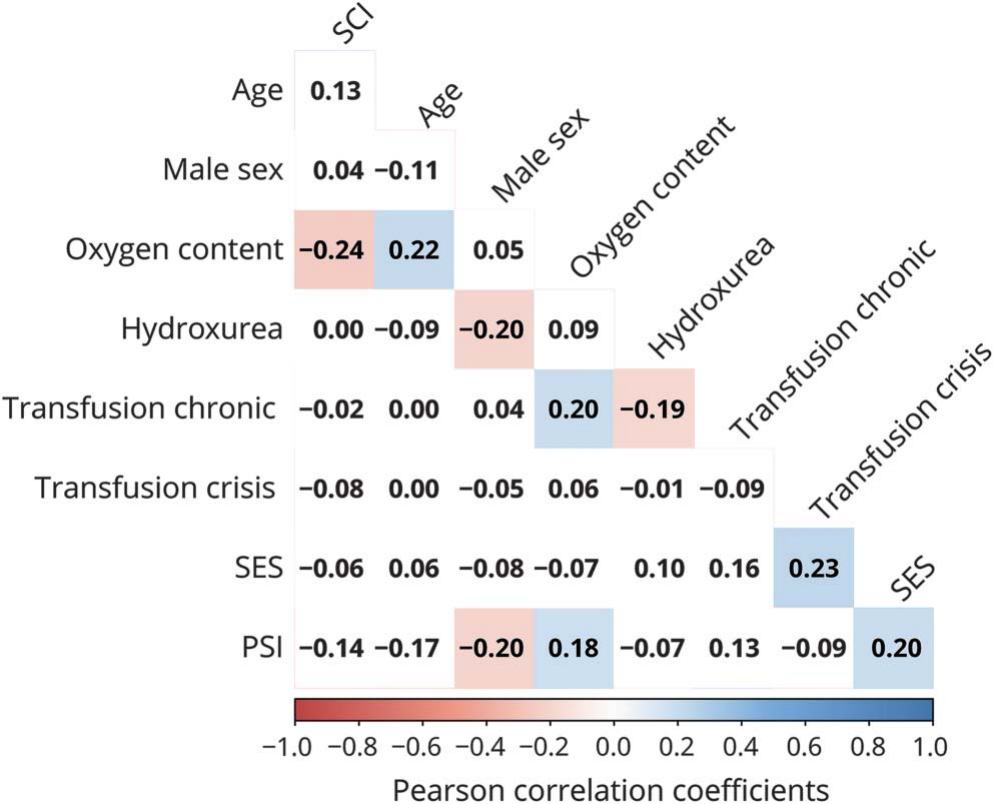
Correlations between predictors of processing speed Correlogram visualizing relationships between variables included in the exploratory regression analysis. Values are zero-order Pearson correlation coefficients. Shaded areas represent significant relationships. Blue colors represent positive relationships, whereas red colors represent negative relationships. Intensity signifies the strength of relationships. PSI = processing speed index; SCI = silent cerebral infarction; SES = socioeconomic status.

Multiple linear regression was conducted to predict PSI from previously implicated variables. Of all the predictors, only male sex, Cao_2_, and education decile (SES) had zero-order correlations with PSI (figure 2). However, in the full model, all predictors apart from SCI and chronic transfusion had partial effects (table 2). The 7-predictor model was able to account for 25% of the variance in PSI (*F*_8,74_ = 3.042, *p* = 0.005, multiple *R*^2^ = 0.25). All predictors remained when 11 influential cases, including all 5 patients on chronic transfusion, were removed from the analysis (*F*_7,64_ = 3.404, *p* = 0.004, multiple *R*^2^ = 0.27).

**Table 2:**
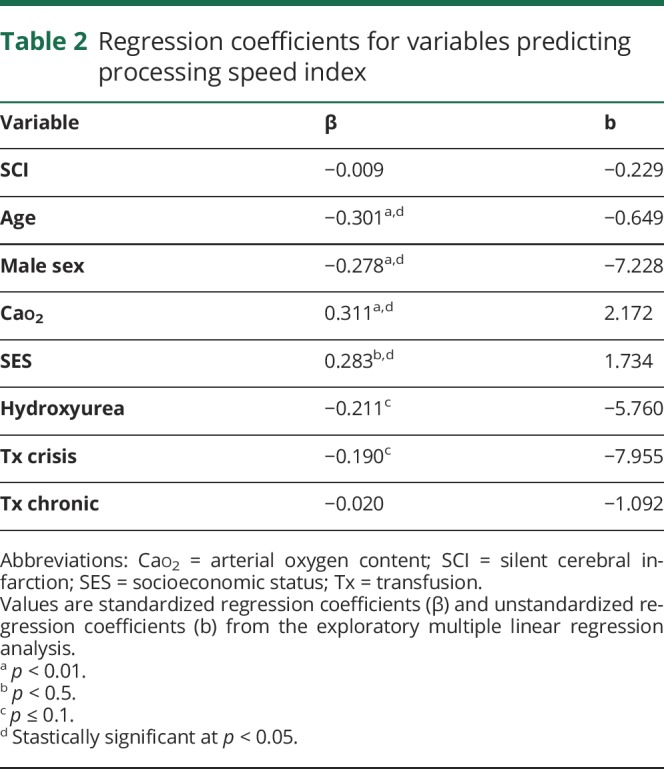
Regression coefficients for variables predicting processing speed index

### Neuroimaging metrics

**Figure 3:**
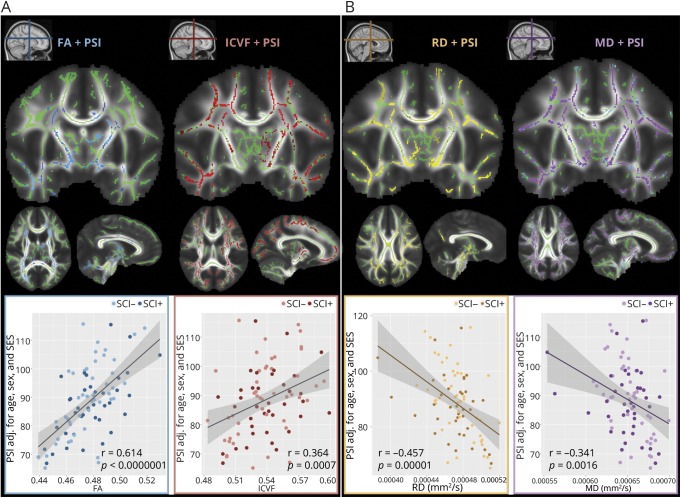
Correlations between diffusion metrics and processing speed (A) Blue voxels indicate areas in which fractional anisotropy (FA) correlated with processing speed index (PSI) (34,392 voxels, *p* < 0.05). Red voxels indicate areas in which intracellular volume fraction (ICVF) correlated with PSI (70,659 voxels, *p* < 0.05). (B) Yellow voxels indicate areas in which radial diffusivity (RD) correlated with PSI (67,296 voxels, *p* < 0.05). Purple voxels indicate areas in which mean diffusivity (MD) correlated with PSI (82,663 voxels, *p* < 0.05). Results were age, sex, education decile (SES), and threshold-free cluster enhancement corrected and overlaid on the group white matter skeleton (green) and the study-specific mean FA template. Adj. = adjusted; SCI = silent cerebral infarction; SES = socioeconomic status.

In patients, PSI was correlated with FA, MD, RD, and ICVF (figure 3). Specifically, decreases in PSI were associated with decreases in FA across the internal capsule and corpus callosum (*r*_81_ = 0.614, *p* < 0.00001, 30,492 voxels), and with decreases in ICVF in more widespread regions covering much of the white matter skeleton, with clusters extending throughout the corpus callosum, corona radiata, and superior and inferior longitudinal fasciculi (*r*_81_ = 0.364, *p* = 0.0007, 70,659 voxels). In addition, decreases in PSI were associated with increases in MD (*r*_81_ = −0.341, *p* = 0.0016, 82,663 voxels) and RD, also in widespread regions, with many clusters located posteriorly, including the posterior corona radiata and splenium of the corpus callosum, respectively (*r*_81_ = −0.457, *p* < 0.00001, 67,296 voxels). Statistical maps can be viewed and explored interactively at neurovault.org/collections/3510/. These correlations remained when examined in SCI+ and SCI− groups separately. There were no relationships between PSI and axial diffusivity or ODI.

## Discussion

This study provides evidence for a relationship between reductions in processing speed and changes in DTI and NODDI parameters in SCA and models risk factors. Patients with SCA showed processing speed deficits, irrespective of presence of SCI. The results suggest that the degree of slower processing speed is related to loss of white matter integrity, and that lower Cao_2_ and SES may be independent risk factors for deficit.

PSI was lower in patients than controls by 9 scaled points. FSIQ was numerically lower by 4 scaled points, but this difference did not reach significance. Differences in PSI were greater than the often-cited 7-point (one-half of an SD) threshold for clinically meaningful differences.^34^ Moreover, although mean PSI in the patient group fell in the low-average range, 28% of patients had PSI scores that fell in the borderline to extremely low ranges (i.e., scores of <80) compared to 6% of controls. Taken together, these results suggest that although there is variability within the SCA population, patients with SCA are at risk of clinically significant cognitive difficulties. Controlling for PSI abolished a numerical difference between patients and controls in FSIQ. These findings extend those of studies with adults^5^ to children with SCA and are consistent with the notion that slower processing may contribute to other cognitive difficulties. Research on aging in the general population has similarly highlighted that fast and efficient information processing may be a prerequisite for higher-order cognitive abilities.^35^

Although cognitive performance was scaled for age, age was a negative predictor of PSI in our regression model. PSI has yet to be examined longitudinally in SCA, but this finding is in agreement with previous reports and may suggest worsening cognitive function with age in SCA.^1,4,36^ While processing speed has been shown to predict academic attainment in typical development,^37^ further work is required to examine the developmental trajectory of PSI in SCA and to investigate the effect of deficits on abilities potentially important for life outcomes.

In patients, after correcting for the effects of age and sex, strong correlations were found between PSI and multiple diffusion-derived indices of white matter microstructure. Correlated regions were widespread, and only partially overlapped with lesions, suggesting that slower processing speed is related to the integrity of normal-appearing white matter in SCA. These results provide evidence that cognitive impairment in SCA is related to white matter integrity using quantitative microstructure parameters from multishell diffusion MRI, and highlight the utility of novel diffusion imaging methods in identifying functionally relevant white matter changes that may not be visible on conventional clinical MRI. The results are in agreement with the notion that processing speed is a domain-general cognitive ability and may suggest that fast and efficient neural processing is dependent on the integrity of many tracts simultaneously.

Our findings extend previous reports of white matter injury in SCA^16,17^ by highlighting possible functional consequences of such injury, and may reflect links between widespread axonal damage, demyelination, and/or disorganization of fibers and slower processing speed. It has been suggested that demyelination can be represented in DTI parameters by decreases in FA and increases in RD with no change in axial diffusivity,^38^ and in NODDI parameters by decreases in ICVF with no change in ODI.^18,39,40^ Our findings are consistent with this pattern. However, MD appeared to be the most sensitive metric, with more than double the number of voxels correlating with PSI than FA. In this sample, therefore, NODDI did not appear to offer improved sensitivity to functionally relevant microstructural changes. Rather, NODDI and DTI metrics were similarly sensitive.

Of note, the histopathologic processes that drive changes in imaging parameters are not well established, and although NODDI overcomes certain specificity issues in DTI, regions with crossing fibers remain problematic. Moreover, NODDI fixes intrinsic diffusivity to an a priori value, and there is scope to further optimize the choice of this value in the model. Before diffusion changes can confidently be referred to as markers of specific microstructural changes, further work comparing diffusion metrics not only to each other, but also to histology measures, is required.

In this sample, 44% of patients were identified with SCI. Lesions were most frequently bilateral and located in the border zones between arterial distributions in the frontal white matter. These findings are consistent with previous studies using similar MRI protocols and criteria for SCI.^41^ Hemoglobin was lower in patients with SCI than in patients reported radiologically as normal, but Spo_2_ was not. Differences in hemoglobin remained after patients on transfusion were removed from the analysis, suggesting more severe anemia in patients with SCI.

However, there were no differences in FSIQ or PSI as a function of SCI, irrespective of age. Similarly, there were no differences between patients with and without SCI in relationships between white matter microstructure parameters and processing speed, and correlated regions only partially overlapped with lesions, confirming the contribution of loss of integrity in normal-appearing white matter to reduced processing speed. These findings accord with recent studies^4,15^ and, taken together, suggest that neither white matter abnormalities nor cognitive deficits are explicable solely by the presence of SCI; other factors, whether psychosocial or disease-related, are likely to be involved.

In patients, education decile was associated with PSI, an indication that this postcode-based index was able to capture some of the variance in SES that may affect cognition. Previous research has similarly highlighted that lower maternal education may be a risk factor for slower processing speed in SCA.^36^ Moreover, in our regression model, education decile was identified as an independent predictor of PSI, further suggesting that socioeconomic and educational deprivation may be risk factors for slower processing speed in patients with SCA.

However, any effects of education decile were controlled for in our comparisons between patients and controls in cognition, and the control participants in this sample were siblings to patients; therefore, SES differences are unlikely to have had a major effect on the difference in PSI reported in the present study. Moreover, in our exploratory model, Cao_2_, age, and male sex were also identified as predictors of PSI, suggesting that there may be multiple causal pathways to cognitive deficits in SCA, in which disease and socioeconomic factors may interact.

One explanation for this pattern of results may be that SCI is associated with acute anemic events,^42^ where inadequate perfusion within the watershed distribution leads to infarction in brain tissue. Acute anemic events are more common in patients with lower steady-state hemoglobin,^41^ perhaps accounting for the relationship between anemia severity and SCI observed here. By contrast, white matter damage that is below the resolution of clinical MRI may be the result of less severe, but sustained, exposure to hypoxia^15^ secondary to compensatory increases in cerebral blood flow^43^ accompanied by reduced cerebrovascular reserve^9^ and increased oxygen extraction fraction.^7,10,11^ This damage may be more diffuse^15,17^ and therefore more functionally significant, potentially explaining the presence of relationships between PSI and diffusion metrics, the absence of relationships with SCI, and the links between both types of damage and anemia severity. The additional identified predictors of PSI are not inconsistent with this explanation, as hydroxyurea use and recent crisis-related transfusion both increase hemoglobin and Spo_2_ and are prescribed more often in patients with greater disease burden, and there is evidence that males have more severe disease courses.^41^ These findings may explain previous discrepancies in the literature and underscore the need for researchers and clinicians to consider the interplay among risk factors as well as potential confounding effects of treatment.

The identification of Cao_2_ as an independent predictor of PSI is consistent with previous reports of relationships between lower Spo_2_ and cognitive impairment^44^ and white matter damage in SCA,^15^ and with reports of processing speed deficits in the general pediatric population with iron-deficiency anemia^45^ and sleep-disordered breathing^46^ as well as in those living at high altitude.^47^ Taken together, these results may suggest improvement of deficits following interventions that target hypoxemic exposure. Overnight respiratory support appears to be safe and viable in children with SCA and is a treatment option that may hold promise.^23^

The study utilized medical records, and there was significant between-patient variation, with time between steady-state full blood count and Spo_2_ measurement to cognitive assessment varying from 1 day to 6 months. There are few data on the stability of these measures over time. Although low Spo_2_ predicts neurologic complications in SCA,^48^ in patients with hemoglobinopathies, right shift of the oxygen dissociation curve and the presence of carboxyhemoglobin and methemoglobin may lead to overestimation.^49^ Daytime and nocturnal Spo_2_ are not necessarily correlated in SCA, with a greater proportion of patients experiencing desaturation at night.^50^ Furthermore, we used postcode rather than direct measures of SES, which were unavailable for the majority. Because of these limitations, we were not able to comprehensively model specific disease and socioeconomic risk factors for slower processing speed.

Further work is required not only to determine risk factors for and mechanisms of white matter injury and cognitive impairment in SCA but also to establish whether the underlying pathology is preventable or reversible. To this end, future work will need to disentangle the effects of SCA pathology and to separate them from the effects of psychosocial factors. As this will require regression and potentially more advanced statistical modeling, future quantitative MRI studies are warranted.

This study provides evidence that reduced processing speed is correlated with widespread white matter abnormalities using quantitative microstructure parameters from multishell diffusion MRI. Although lesion status is frequently used as a proxy of disease severity, the results from this study indicate cognitive difficulties in the absence of SCI and highlight the consequences of possible damage to normal-appearing white matter. Clinicians should therefore assess for cognitive difficulties irrespective of presence of SCI, and future research should utilize diffusion MRI as a tool to further investigate potential mechanisms of cognitive impairment in SCA as well as to monitor therapies designed to ameliorate cognitive dysfunction. This study adds to a growing body of evidence indicating imaging abnormalities and cognitive impairment that may worsen with age in SCA, which together highlight the need to investigate the effect of early treatment delivery.

## Glossary

Cao_2_: arterial oxygen content
DTI: diffusion tensor imaging
FA: fractional anisotropy
FLAIR: fluid-attenuated inversion recovery
FSIQ: Full Scale IQ
FSL: FMRIB's Software Library
ICVF: intracellular volume fraction
MD: mean diffusivity
NODDI: neurite orientation dispersion and density imaging
ODI: orientation dispersion index
POMS: Prevention of Morbidity in Sickle Cell Disease
PSI: processing speed index
RD: radial diffusivity
SAC: Sleep and Asthma Cohort follow-up study
SCA: sickle cell anemia
SCI: silent cerebral infarction
SES: socioeconomic status
Spo_2_: daytime oxygen saturation
TE: echo time
TR: repetition time
WAIS: Wechsler Adult Intelligence Scale
WASI: Wechsler Abbreviated Scale of Intelligence
WISC: Wechsler Intelligence Scale for Children
